# IgG4-related disease with biopsy confirmed inflammatory polyneuropathy

**DOI:** 10.1093/rap/rkae101

**Published:** 2024-08-27

**Authors:** Nehaal Ahmed, Michael Skolka, Matthew J Koster

**Affiliations:** Division of Internal Medicine, Mayo Clinic, Rochester, MN, USA; Department of Neurology, Mayo Clinic, Rochester, MN, USA; Department of Rheumatology, Mayo Clinic, Rochester, MN, USA

Key message

- Peripheral neuropathy with biopsy confirmed inflammatory neuropathy is an exceedingly rare manifestation of IgG4-related disease.

Dear Editor, Presentations of IgG4-related disease (IgG4-RD) can be highly variable. Providers should be aware of atypical features. We describe a patient with IgG4-RD with biopsy-confirmed inflammatory polyneuropathy.

A 73-year-old male with history of occupational asbestos exposure presented with migratory pain in the upper extremities, back and left flank two weeks after hernia surgery. CT of the lumbar and thoracic spine demonstrated a right lower lobe pleural-based lung mass (Supplementary Fig. S1, available at *Rheumatology Advances in Practice* online) and bilateral perinephric stranding (Supplementary Fig. S2, available at *Rheumatology Advances in Practice* online). Levofloxacin was prescribed for presumed urinary tract infection and flank pain improved. At the end of the levofloxacin course, he developed rapidly progressive neuropathy with lower extremity discomfort, numbness and weakness resulting in wheelchair dependency.

Evaluation with PET-CT demonstrated uptake in the pleural mass (SUV Max 4.6), perirenal spaces and periaortic infrarenal abdominal aorta with periaortic soft tissue extending to the iliac arteries (SUV Max 3.6) (Supplementary Fig. S3, available at *Rheumatology Advances in Practice* online). Notable laboratory findings (Supplementary Table S1, available at *Rheumatology Advances in Practice* online) included an ESR of 90 mm/h, CRP of 10.3 mg/l, and IgG4 level of 454 mg/dl (ref range normal 2–121).

Biopsy of the lung mass demonstrated fibrosis with patchy inflammation and mild increase in IgG4 cells. Scattered histiocytes were observed that were CD163(+) but BRAFV600E(−) (Supplementary Fig. S4, available at *Rheumatology Advances in Practice* online).

Nerve conduction studies demonstrated a severe, length dependent, axonal predominant peripheral neuropathy with superimposed right L5 radiculopathy and right ulnar mononeuropathy. MRI of the brain was negative for pachymeningitis. MRI of the spinal cord noted non-specific enhancement of the cauda equina without nodularity or enlargement. Cerebrospinal fluid analysis demonstrated elevated total nucleated cell count of 13 cells/mcL, markedly increased protein at 439 mg/dl (ref range 0–35), normal glucose at 108 mg/dl, and elevated IgG at 57.9 mg/dl (ref range <8.1 mg/dl). CSF Lyme, autoimmune and paraneoplastic panel, and cytology were negative. Sural nerve biopsy noted axonal degeneration, mildly decreased myelinated fibres and multiple epineural perivascular inflammatory collections with rare, atypical lymphocytes. No histiocytes were noted (Fig. 1).

**Figure 1.**
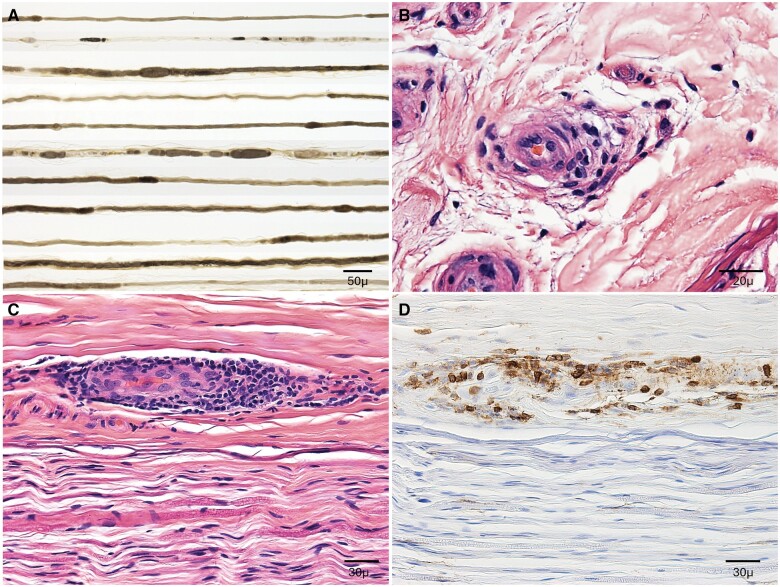
Nerve biopsy. Left sural nerve biopsy showed evidence of an inflammatory neuropathic process. (A) Teased fibre preparation revealed axonal degeneration, segmental remyelination and myelin reduplication. (B) Haematoxylin and eosin (H&E) stained transverse section revealed a small epineural perivascular mononuclear inflammatory cell collection. (C) H&E stained longitudinal section revealed a large epineural perivascular mononuclear inflammatory cell collection that (D) stains positive for CD-45, lymphocyte common antigen

To exclude alternative diagnoses, biopsy of the perinephric stranding was pursued which demonstrated fibroadipose tissue with mild chronic inflammation and mild increase in IgG4. Scattered histiocytes were again noted which were BRAF V600E(−) (Supplementary Fig. S5, available at *Rheumatology Advances in Practice* online). Whole body bone scan was negative for metadiaphyseal uptake.

The patient was treated with three days of intravenous methylprednisolone 1000 mg, followed by 6 weekly doses. He received two doses of rituximab, 1000 mg separated by two weeks, and was tapered off glucocorticoids. Three months later he had resolution of polyneuropathy and weakness. At one year follow up he remained in remission with IgG4 level normal at 42.5 mg/dl.

IgG4-related peripheral neuropathy is exceedingly rare, occurring in less than 1% of patients [1]. It is characterized by lymphoplasmacytic infiltration and fibrosis of peripheral nerves with a predilection for the epineurium, resulting in axonal damage and decreased myelinated fibres, typically without concurrent demyelination [2, 3]. It rarely occurs as an isolated phenomenon without other organ involvement [3]. Given lymphoplasmacytic infiltration is not always observed and signs of perivascular inflammation can be present on nerve biopsy, the aetiology of neuropathy may be falsely attributed to other causes, such as vasculitis or paraneoplastic neuropathy [4, 5].

In this case, features concerning for the possibility of Erdheim-Chester Disease (ECD), a non-Langerhans cell histiocytic disorder that can mimic IgG4-RD, include the presence of periaortic soft tissue with concomitant involvement of perirenal adipose tissue and peripheral axonal neuropathy, as they are more common in ECD [6–8]. Furthermore, ECD can demonstrate non-specific elevation of serum IgG4 and variable degree of lymphoplasmacytic infiltrate and fibrosis in lesional tissue. Although PET-CT has modest sensitivity for osteosclerotic lesions, bone scan is more specific [6]. While the presence of characteristic ‘foamy’ histiocytes with map kinase mutations distinguish ECD from IgG4-RD, biopsies from diagnostically ideal locations (e.g. perirenal) may have low levels or absence of pathologic histiocytes in fibrosis predominant samples [6, 8]. In this case, ECD was ultimately excluded and the patient was treated as IgG4-RD given the overall clinical, radiologic, and serologic evidence suggestive of IgG4-RD, lack of characteristic findings of ECD on multiple tissue samples and negative bone scan.

In conclusion, inflammatory polyneuropathy is a rare manifestation of IgG4-RD. Providers should be aware of this uncommon co-morbidity as correct identification and prompt treatment may result in symptom resolution, as was achieved in this case.

## Supplementary Material

rkae101_Supplementary_Data

## Data Availability

The data underlying this article are available in the article and in its online supplementary material.
