# Supplementary material for: IgG4-related disease with biopsy confirmed inflammatory polyneuropathy
Source: Rheumatol Adv Pract. 2024 Aug 27;8(3):rkae101. doi: 10.1093/rap/rkae101 (PMC11374030; doi:10.1093/rap/rkae101)
Supplement: rkae101_Supplementary_Data [file rkae101_supplementary_data.docx]

Supplementary material


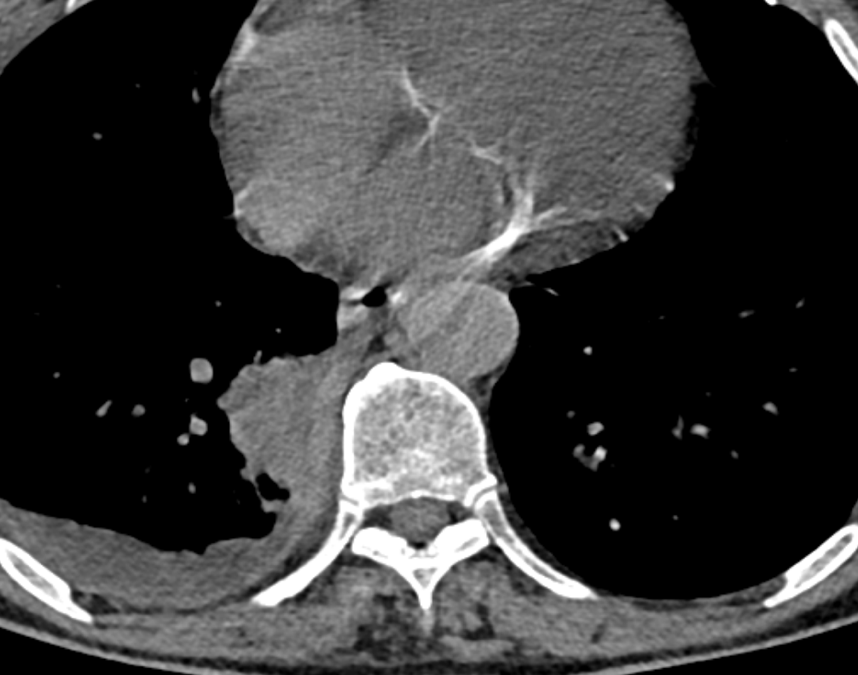

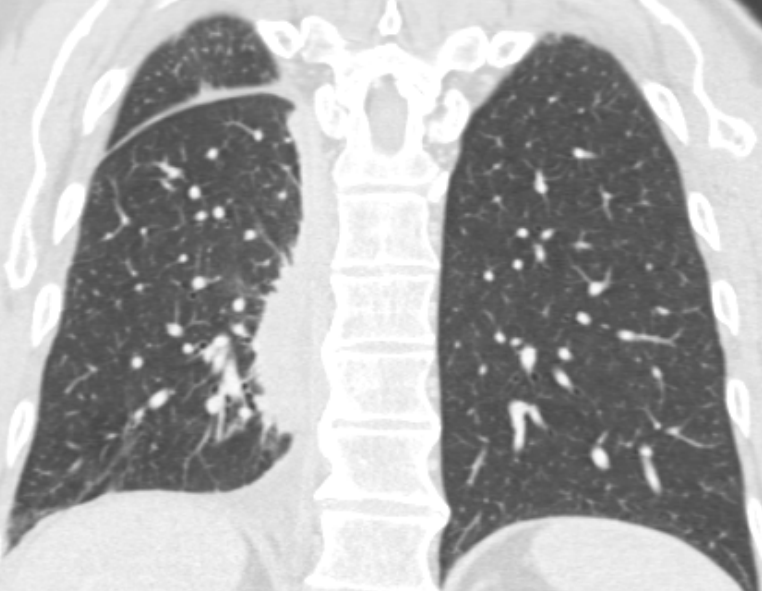
Supplementary Figure S1: Lung Mass

Left image: CT Thoracic Spine Coronal Slice incidentally demonstrating right lower lobe pleural based mass with small loculated effusion and left lower lobe calcified pleural plaque. Right image: CT Chest Axial Slice redemonstrating right lower lobe pleural based mass with small loculated pleural effusion.


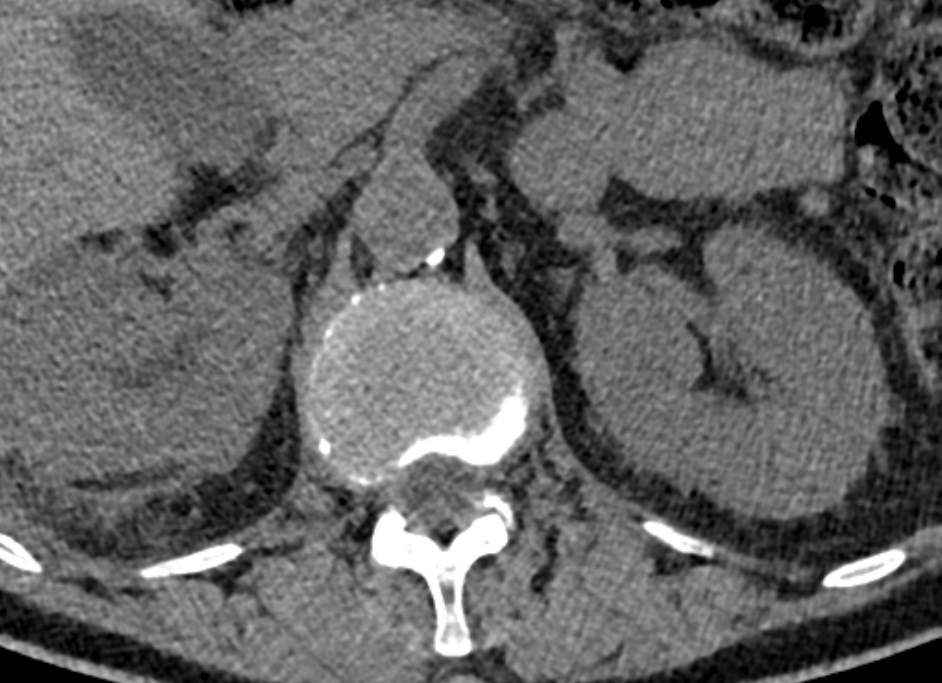
Supplementary Figure S2: Bilateral Perinephric Stranding

CT Lumbar Spine Axial Slice demonstrating incidentally noted nonspecific bilateral perinephric stranding.


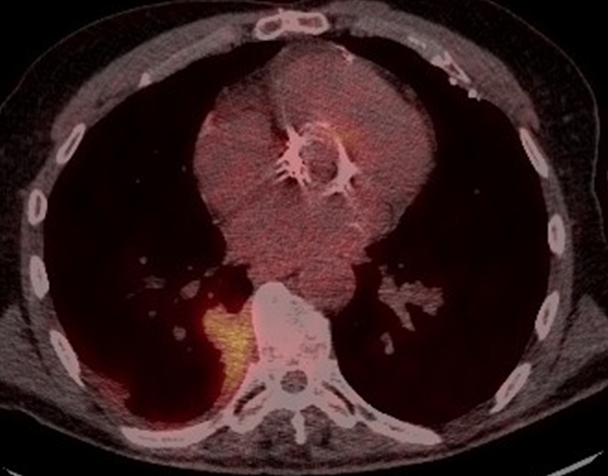
Supplementary Figure S3: PET-CT

Upper Image: PET-CT Axial slice demonstrating right pleural mass with mild-to-moderate uptake (SUV Max 4.6).

Middle Image: PET-CT Axial slice demonstrating mild-to-moderate uptake in the bilateral perirenal spaces (SUV Max 3.6).


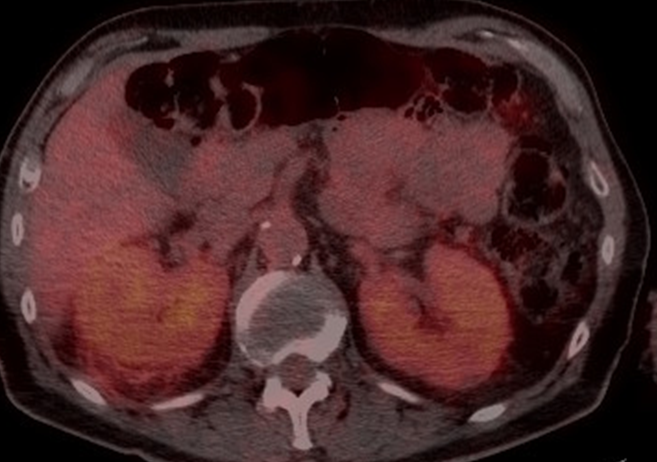
Bottom Image: PET-CT Axial Slice demonstrating mild-to-moderate uptake in the periaortic soft tissue surrounding the infrarenal abdominal aorta (SUV Max 3.6).


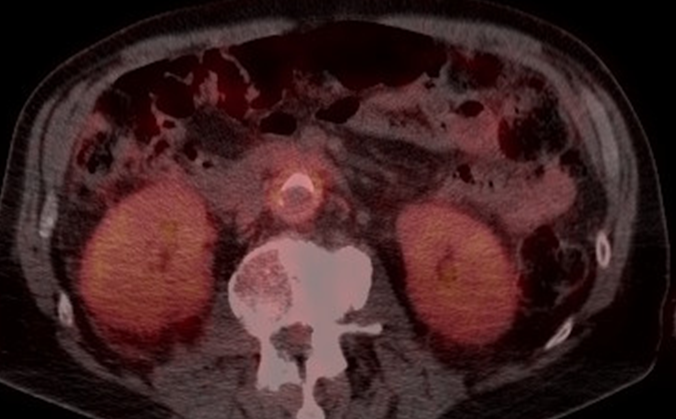


Supplementary Table S1: Laboratory Findings

| **Test** | **Result** | **Reference Value** | **Reference Unit** |
| --- | --- | --- | --- |
| ANA by ELISA | 2.1 (weakly positive) | 1.1-2.9 weak positive | - |
| ANA by Hep2 | 1:160 (positive) | < 1:80 | titer |
| Rheumatoid Factor | 56 | < 15 | IU/mL |
| Cyclic Citrullinated Peptide Ab, S | < 15.6 (negative) | < 20.0 | - |
| Double Stranded DNA Ab, IgG, S | < 12.3 | < 30.0 | IU/mL |
| SS-A/RO Ab, IgG, S | < 0.2 | < 1.0 | - |
| SS-B/La Ab, IgG, S | < 0.2 | < 1.0 | - |
| Smith Ab, IgG, S | < 0.2 | < 1.0 | - |
| RNP Ab, IgG, S | 0.7 | < 1.0 | - |
| Scl 70 Ab, IgG, S | < 0.2 | < 1.0 | - |
| Jo 1 Ab, IgG, S | < 0.2 | < 1.0 | - |
| Myeloperoxidase Ab, (MPO), S | < 0.2 | < 0.4 | - |
| Proteinase 3 Ab (PR3), S | < 0.2 | < 0.4 | - |
| ESR | 90 | 3-28 | mm/hr |
| CRP | 10.3 | < 5.0 | mg/L |
| IgG4, Ig Subclasses | 454.1 | 2.4-121.0 | mg/dL |
| Hemoglobin A1c, B | 5.9% | 4.0-5.6% | - |
| TSH, Sensitive | 1.1 | 0.3-4.2 | mIU/L |
| SPEP | Polyclonal Hypergammaglobulinemia | - | - |

ANA: Antinuclear antibody; ELISA: enzyme-linked immunosorbent assay; SS-A: anti-Sjogren’s syndrome related antibody A; SS-B: anti-Sjogren’s syndrome related antibody B; RNP: anti-Ribonucleoprotein antibody; Scl 70: anti-Scleroderma 70 kD extractable immunoreactive fragment antibody; ESR: Erythrocyte Sedimentation Rate; CRP: C-reactive protein; IgG4: Immunoglobulin G4; Ig: Immunoglobulin; A1c: glycated hemoglobin; TSH: Thyroid stimulating hormone; SPEP: Serum protein electrophoresis


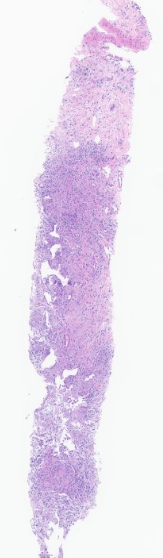

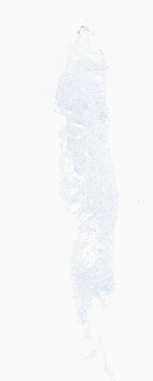

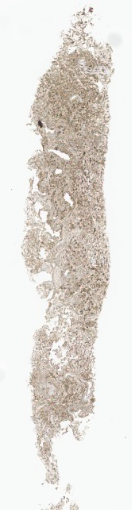

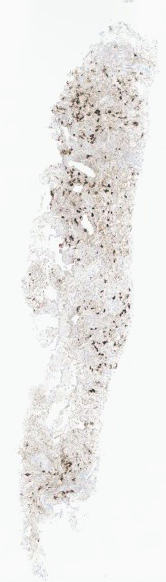
Supplementary Figure S4: Lung Biopsy

Biopsy of the lung mass demonstrating fibrosis with patchy inflammation and mild increase in IgG4 cells (20/hpf). Scattered histiocytes observed that are CD163(+) but BRAFV600E(-). (Left image hematoxylin and eosin staining; left middle image IgG staining; right middle image IgG4 staining; right image BRAFV600E staining; magnification 40x).


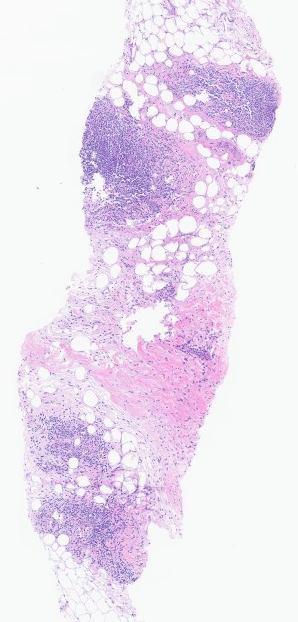

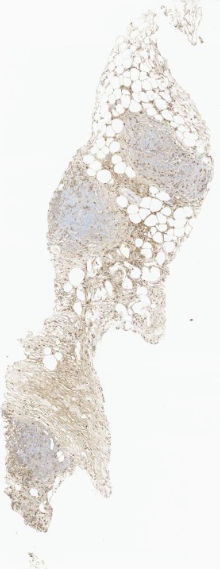

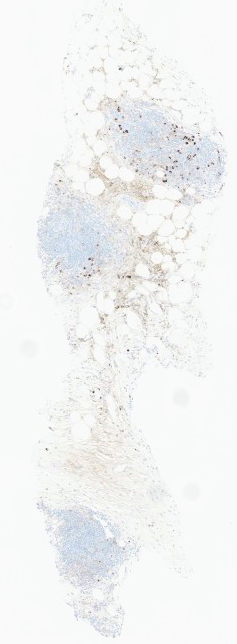

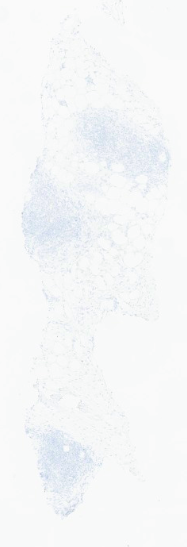
Supplementary Figure S5: Perinephric Stranding Biopsy

Biopsy of perinephric stranding demonstrating fibroadipose tissue with mild chronic inflammation and mild increase in IgG4 (10/hpf). Scattered histiocytes were noted that were BRAF V600E negative. (Left image hematoxylin and eosin staining; left middle image IgG staining; right middle image IgG4 staining; right image BRAFV600E staining; magnification 40x).
